# Identification of hypervirulent *Klebsiella pneumoniae* carrying *terW* gene by MacConkey-potassium tellurite medium in the general population

**DOI:** 10.3389/fpubh.2022.946370

**Published:** 2022-08-24

**Authors:** Xiufeng Wu, Fuguo Zhan, Jiawei Zhang, Shanjian Chen, Bin Yang

**Affiliations:** ^1^Department of Laboratory Medicine, Fujian Medical University Union Hospital, Fuzhou, China; ^2^Department of Laboratory Medicine, Gene Diagnosis Research Center, The First Affiliated Hospital, Fujian Medical University, Fuzhou, China; ^3^Fujian Key Laboratory of Laboratory Medicine, The First Affiliated Hospital, Fujian Medical University, Fuzhou, China; ^4^First Clinical College, Fujian Medical University, Fuzhou, China

**Keywords:** hypervirulent *Klebsiella pneumoniae*, *terW* gene, potassium tellurite resistance, epidemic surveillance, general population

## Abstract

**Objectives:**

To establish a MacConkey-potassium tellurium medium-based method for selectively culturing *terW* gene-positive *Klebsiella pneumoniae* (KP), to evaluate its performance and apply it to identifying particular clonal hypervirulent KP (hvKP) strains in epidemiological surveillance.

**Methods:**

The virulence genes, *rmpA, iutA*, and *terW*, were detected by PCR. The minimum inhibitory concentration of potassium tellurite of hvKP (*rmpA*^+^/ *iutA*^+^) and classical KP (*rmpA*^−^ and *iutA*^−^) was determined using the agar dilution method. The MacConkey medium containing 4 μg/ml potassium tellurite was prepared and the performance in detecting *terW*^+^ KP was evaluated, including an agreement with PCR and positive/negative predictive value. Fecal samples from healthy volunteers in Fujian were collected and cultured in the medium, then positive strains were identified using MALDI-TOF MS, antimicrobial susceptibility was tested by Kirby-Bauer assays, and virulence genes and capsular serotype genes were tested by PCR.

**Results:**

In KP isolated from clinical specimens (*N* = 198), the positive rate of *terW* was 37.9%, and the detection rate of *terW* in hvKP was significantly higher than that in classical KP (70.6% *vs* 13.3%). The potassium tellurite resistance levels of *terW*^+^ (*N* = 75) and *terW*^−^ (*N* = 55) KP were 8–128 μg/ml and <1–8 μg/ml, respectively, with significant differences. KP was selectively cultured on a MacConkey medium with 4 μg/ml potassium tellurite, and its agreement with PCR was good (Kappa=0.936), and the positive and negative percent agreement and positive and negative predictive values were 100% (75/75), 92.7% (51/55), 94.9% (75/79), and 100% (51/51), respectively. The prevalence of tellurite-resistant KP was 16.7% (86/516) in fecal samples from healthy volunteers, among which the positive rate of *terW* was 100% (86/86). The antimicrobial resistance characteristics of *terW*^+^ KP showed no difference between healthy volunteers and inpatients. The most common capsular serotypes associated with high virulence were K1, K2, and K57.

**Conclusions:**

The MacConkey medium containing 4 μg/ml potassium tellurite could easily select and culture *terW*^+^ KP in fecal samples with high sensitivity and specificity, which is a practical method for the epidemic surveillance of hvKP in the general population.

## Introduction

According to the 2018 China Antimicrobial Surveillance Network, *Klebsiella pneumoniae* (KP) has become the second most common gram-negative bacillus isolated in clinics (1). In 1986, Taiwanese researchers first reported a KP that can cause multi-site abscesses and defined it as hypervirulent *K. pneumoniae* (hvKP) (2). Since then, hvKP has been reported in most countries and regions around the world, especially in Asia, where China is a high-incidence area of hvKP (3–5). The hvKP tends to show more virulence than traditional infections caused by classic *K. pneumoniae* (cKP), and it is more likely to cause community-acquired invasive infections in younger healthy individuals with distant metastases in 11 to 80% of cases, such as brain, eye, spleen, and prostate, and it even results in severe infection and high mortality (about 3–32%) (6). Given the high pathogenicity of this type of KP and its genetic susceptibility in Asian populations, epidemiological studies are necessary to clarify the prevalence and distribution of hvKP.

Researchers found that abscesses caused by KP were related to their carrying *terW-iutA-rmpA-silS* gene-derived locus (7, 8). Therefore, abscesses caused by KP often require *rmpA, iutA, terW*, and *silS* genes to work together. It is known that the *rmpA* gene is an important regulator of the extracellular polysaccharide synthesis capsule forming mucous colonies (9–11), and the *iutA* gene is a virulence gene responsible for encoding aerobactin siderophore (11, 12). The *silS* gene is mainly related to silver resistance (13), and the *terW* gene is mainly related to tellurium resistance (14–16). Most hvKp were able to reduce tellurite and form black colonies due to the presence of a major virulence plasmid containing a tellurite resistance gene. Passet et al. found that the *terW*^+^ KP was closely related to the three clonal bacterial groups CG23, CG65, and CG86 that most commonly cause community-acquired purulent infections (16). Although the mechanism of tellurite resistance has not been clearly studied, it is generally considered to be an important virulence factor in highly pathogenic bacteria (17). Some researchers have found that the presence of the *ter* operon can increase the oxidative stress response mediated by hydrogen peroxide, which can resist the action of reactive oxygen species, increase the tolerance to tellurite, resist lysosomal oxidation, and enhance bacterial escape. The primary immune response of the host increases the virulence of specific strains by prolonging their ability to survive in macrophages and neutrophils (17–20). The above studies have shown that the *terW*^+^ KP is closely related to specific hvKP strains circulating in the community, and most of them exhibit potassium tellurite resistance.

The intestinal tract is one of the major reservoirs for KP, and most of the infections caused by KP are related to previous intestinal colonization (21–24). So the intestinal colonization of KP is an important factor in causing subsequent parenteral infection. Most of the previous studies on intestinal KP colonization were hospitalized patients, and there were few studies on KP colonization in healthy people. Since purulent infections caused by hvKP are more common in young healthy individuals without underlying diseases, and most of them are community-acquired infections (25), epidemiological investigations are of great importance for tracing the source of purulent infections and treating them, such as understanding the colonization rate, molecular characteristics and resistance patterns of *terW*^+^ KP to common antibiotics in the healthy population (26).

At present, the detection of *terW* by PCR can be used for clinically monitoring the virulence of KP, and it has good sensitivity. However, PCR detection of *terW* gene requires operations such as separation of pure strains and extraction of bacterial genomic DNA, which also has high requirements for instruments, so it is not suitable for primary laboratories and epidemic investigation. Since the previous epidemiological investigating method is labor-consuming and time-costing (27), it is necessary to develop an economical and convenient method for epidemiological investigation, to help trace the virulence development and prevent infection in the community and nosocomium. As we know, the MacConkey medium is a commonly used selective medium for the isolation of KP from fecal specimens. Therefore, a certain concentration of potassium tellurite based on MacConkey medium can be used to selectively culture *terW*^+^ KP.

In this study, based on the characteristics of *terW* gene with tellurite resistance, we verified the relationship between *terW* gene and tellurite resistance, evaluated the performance of the tellurite medium to detect *terW*^+^ KP, and applied it to investigate healthy people. This study aims to provide a novel perspective and practical method for the epidemic surveillance of hvKP in the general population.

## Materials and methods

### Sample collection and the definition of hvKP

A total of 198 non-repeated KP strains isolated from clinical specimens of inpatients in the First affiliated hospital of Fujian Medical University from June 2018 to December 2018 were collected. All bacterial isolates were identified using standard biochemical laboratory methods. Identified colonies were scraped with dry filter paper, put in a 1.5 ml sterile tube, and stored at -80°C.

From September 2019 to November 2019, 516 non-repetitive fresh fecal specimens from healthy volunteers were collected. Volunteers provided fecal specimens for this study after informed consent, and eligible volunteers were selected by asking about medical history and travel history. Briefly, these healthy volunteers were individuals aged 18–59 years, male or female, without acute infectious diseases or a history of using antibiotics within 4 weeks, and they all settled in Fujian, China, and did not travel to other provinces within 1 year.

Previous studies concluded that the presence of *rmpA* and *iutA* for hvKP identification was > 0.95 (11, 28), so *rmpA* and *iutA* were often used to define hvKP (5, 28–31). In this study, KP with *rmpA* or *iutA* was defined as hvKP (*rmpA*^+^/ *iutA*^+^), including the case where both genes were positive at the same time, while KP with neither positive *rmpA* nor *iutA* was defined as cKP (*rmpA*^−^ *and iutA*^−^).

### Detection of *rmpA, iutA, terW* gene by PCR

Bacterial DNA was extracted by boiling method. Primers were designed according to previous reports (8, 32). The amplification conditions were in accordance with the instructions of the PCR amplification kit (TaKaRa, Japan).

About 5 μl of amplified products were separated by 2% agarose electrophoresis at a voltage of 90 V for 30 min. Gels were exposed by the imaging system to interpret results qualitatively. The positive product was sent to Shanghai Sangon Biotech for sequencing confirmation.

### Preparation of MacConkey-potassium tellurite medium

To prepare MacConkey-potassium tellurite (MCKT) medium, MacConkey medium powder (Qingdao Hope Bio, Batch Number: 20180622) was prepared according to the instructions and added with different amounts of 1 % potassium tellurite (Qingdao Hope Bio, Batch Number: 20180703) solution to prepare MacConkey medium containing potassium tellurite at concentrations of 1, 2, 4, 8, 16, 32, 64, 128, 256, and 512 μg/ml.

The KP strains stored at −80°C were transferred to a Columbia blood medium and incubated at 35°C for 24 h. After re-identification and confirmation, three to four colonies were picked and placed in 5 ml of sterilized saline to prepare a bacterial suspension with a McFarland turbidity concentration of 0.5 (1 × 10^8^ CFU/ml). After shaking and mixing, we added 10 μl of the bacterial suspension and spread it evenly on a MacConkey medium containing different concentrations of potassium tellurite. After 24 h of incubation at 35°C, the results were observed. The lowest concentration of potassium tellurite that inhibited the growth of the strain was the potassium tellurite Minimum Inhibitory Concentration (MIC). Colony growth would occur when pink or black colonies were observed on the MCKT medium. Three replicate experiments were performed for each strain, and the potassium tellurite MIC was finally determined.

### Performance of the MCKT medium in detecting *terW*^+^ KP

According to PCR results, clinical sample-derived KPs are classified as *terW*^+^ KP strains or *terW*^−^ KP strains. The KP with a negative *terW* gene and potassium tellurite resistance level of 180 μg/ml was the negative quality control strain and KP with a known negative *terW* gene and potassium tellurite resistance level of <1μg/ml was identified as the negative quality control strain.

#### Consistency of MCKT medium culture method compared with PCR

After re-identification and confirmation, a single colony was scraped to prepare 0.5 McFarland turbidity bacteria suspension (1 × 10^8^ CFU/ml) with sterilized ddH2O. A loop of about 10 μl of the suspension was taken and streaked on the MCKT medium. After incubation at 35°C for 24 h, colony growth occurred when pink or black colonies were observed on the MCKT medium. Three replicates were performed for each strain. Using the PCR results as the “gold standard”, the positive percent agreement (PPA), negative percent agreement (NPA), negative predictive value, and positive predictive value of MCKT medium were calculated, respectively. The consistency of the method for detecting *terW*^+^ KP with PCR was also evaluated.

#### Lower limit of detection of MCKT medium

All *terW*^+^ KPs isolated from 198 non-repeated KPs were included. These strains were cultured in Columbia blood medium, followed by identification and confirmation, 3 to 5 colonies were used to prepare a bacterial suspension at a McFarland turbidity concentration of 0.5 (1 × 10^8^ CFU/ml), and a series of 10-fold dilutions were performed to obtain KP suspensions of different concentrations (10^1^ CFU/mL~10^7^ CFU/ml), and 1 ml suspensions of different concentrations were taken, and it was cultured at 35°C for 24 h. Colony growth would occur if pink or black colonies were observed on the medium. Three replicates were performed for each strain.

### Identification of cultured microbes by MALDL-TOF

About 0.1 g of fecal sample from each healthy volunteer was inoculated on the MCKT medium and cultured overnight in an aerobic incubator at 35°C. The bacteria were identified using the MALDI-TOF mass spectrometer (Bruker Daltonique, Germany) according to the instructions. MALDI Biotyper 2.3 database was used for strain map comparison, and the score ≥2.0 indicated that the sample mass spectrum was highly similar to the corresponding strain map in the database and the result was credible.

### Antimicrobial susceptibilities assay

VITEK-2 automatic microbiological analyzer (French bio Mérieux) performs antimicrobial susceptibility test on 198 clinical samples according to the instrument and reagent instructions.

A total of 86 isolates of *terW*^+^ KP isolates from samples of healthy volunteers and 75 isolates from clinical samples were used to detect KP by Kirby-Bauer disc diffusion method for susceptibilities assay to ampicillin, cefazolin, piperacillin/tazobactam, ceftriaxone, cefepime, imipenem, meropenem, ciprofloxacin, and tobramycin. *E. coli* ATCC25922 was used as the quality control strain, and the interpretations of the results were carried out in accordance with the 2019 edition of the CLSI antimicrobial susceptibility test implementation standards (33).

### Capsular serotype molecular detection

The molecular characteristics of capsular serotypes were detected by PCR, the primers were designed according to previous reports (31, 34–36), and the rest of the PCR steps were the same as above.

### Statistical analysis

Statistical analysis and graphing were performed with SPSS19.0 and GraphPad Prism 7.0 software. The chi-square test was used to compare the rates. The differences in the resistance levels of KP to potassium tellurite between the two groups were analyzed by the Mann–Whitney U test (two-sample rank-sum test). *p* < 0.05 was considered statistically significant.

## Results

### The relationship between *terW* and hvKP

#### The characteristics of cKP and hvKP in aspects of genes and resistance

The *iutA, rmpA*, and *terW* genes were detected in 40.9% (81/198), 40.4% (80/198), and 37.9% (75/198) of KP isolated from inpatients' samples, respectively. The *terW* was shown to be present in 70.6% (60/85) hvKP and 13.3% (15/113) cKP, respectively. The detection rate of *terW* in hvKP was significantly higher than that in cKP (*p* < 0.001). Besides, the hvKP and cKP differ from each other significantly in the aspect of antibiotic resistance (Table 1).

**Table 1:** The antibiotics resistance of KP isolated from inpatients.

**Antibiotic**	**cKP** ***n =* 113(%)**	**hvKP** ***n =* 85(%)**	***p*-Value**
Ampicillin	113 (100.0)	85 (100.0)	/
Ampicillin/sulbactam	48 (42.5)	14 (16.5)	<0.001
Piperacillin	43 (38.1)	14 (16.5)	0.001
Piperacillin/tazobactam	30 (26.5)	2 (2.4)	<0.001
Cefazolin	50 (44.2)	10 (11.8)	<0.001
Cefuroxime	45 (39.8)	14 (16.5)	<0.001
Cefuroxime axetil	46 (40.7)	14 (16.5)	<0.001
Ceftriaxone	43 (38.1)	6 (7.1)	<0.001
Ceftazidime	42 (37.2)	2 (2.4)	<0.001
Cefepime	29 (25.7)	1 (1.2)	<0.001
Imipenem	25 (22.1)	0 (0.0)	<0.001
Meropenem	25 (22.1)	0 (0.0)	<0.001
Levofloxacin	31 (27.4)	4 (4.7)	<0.001
Ciprofloxacin	32 (28.3)	5 (5.9)	<0.001

KP, Klebsiella pneumoniae; hvKP, Hypervirulent KP; cKP, Classic KP.

#### Relationship between terW gene and tellurite resistance

All 75 *terW*^+^ KP and 55 randomly selected *terW*^−^ KP was tested for potassium tellurite resistance. The potassium tellurite resistance level of *terW*^+^ KP strains (*n* = 75) ranged from 8 to 128 μg/ml. And the resistance level of *terW*^−^ KP strains (*n* = 55) was ranged from <1 to 8 μg/ml (Table 2). The potassium tellurite resistance level of *terW*^+^ KP was significantly higher than that of *terW*^−^ KP (*p* < 0.001).

**Table 2:** The presence of *terW* gene of KP isolated from inpatients' samples and their MIC of potassium tellurite.

**MIC value of potassium tellurite (ug/ml)**	***terW^+^*** **(%, *n =* 75)**	***terW^−^*** **(%, *n =* 55)**
<1	0 (0)	45 (81.8)
2	0 (0)	5 (9.1)
4	0 (0)	1 (1.8)
8	1 (1.3)	4 (7.3)
16	2 (2.7)	0 (0)
32	23 (30.7)	0 (0)
64	42 (56.0)	0 (0)
128	7 (9.3)	0 (0)
256	0 (0)	0 (0)
512	0 (0)	0 (0)

KP, Klebsiella pneumoniae; MIC, Minimum Inhibitory Concentration.

According to the potassium tellurite resistance level of KP, to ensure the growth of *terW*^+^ KP, and to inhibit the growth of *terW*^−^ KP to the greatest extent, the final concentration of potassium tellurite was set at 4μg/ml in the MCKT medium.

### The performance of MCKT medium in detecting *terW*^+^ KP

#### Agreement values between the MCKT medium culturing method and PCR in detecting *terW*^+^ KP

In detecting *terW* gene, PCR was used as a comparing method. For MCKT medium containing 4 μg/ml potassium tellurite in selectively culturing *terW*^+^ KP, the positive percent agreement was 100% (75/75), the negative percent agreement was 92.7% (51/55), the positive predictive value was 94.9 % (75/79) and the negative predictive value was 100% (51/51) (Table 3). The Kappa value between the MCKT medium culturing method and PCR was 0.936 (*p* < 0.05), which indicated that the consistency was good.

**Table 3:** Clinical performance comparison of MCKT medium and PCR in detecting *terW*^+^ KP.

**MCKT medium method**		**PCR method**	**Total number**
	**Positive (*n =* 75)**	**Negative (*n =* 55)**	
Positive	75	4	79
Negative	0	51	51

MCKT, MacConkey-potassium tellurite; PCR, polymerase chain reaction; KP, Klebsiella pneumoniae.

#### Lower limit of detection of MCKT medium in detecting *terW*^+^ KP

A total of 75 strains of *terW*^+^ KP were prepared as serial dilution concentration suspensions (10^1^~10^7^ CFU/ml), and 1 ml of the suspension was inoculated on an MCKT medium containing 4 μg/ml potassium tellurite. The minimum amount of bacteria that can be grown was 10^1^ ~ 10^3^ CFU/ml (61 strains of 10^1^ CFU/ml, 13 strains of 10^2^ CFU/ml, and 1 strain of 10^3^ CFU/ml). When inoculated with 10^1^ CFU/ml, 81.3% (61/75) of *terW*^+^ KP could grow on this medium (Table 4).

**Table 4:** Minimum concentration of bacteria that can grow in MCKT medium.

**Concentration of *terW*^+^ KP (CFU/mL)**	**10^7^**	**10^6^**	**10^5^**	**10^4^**	**10^3^**	**10^2^**	**10^1^**
Number of strains	0	0	0	0	1	13	61

MCKT, MacConkey-potassium tellurite; KP, Klebsiella pneumoniae.

### Verification of the MCKT medium culture method in the general population

#### The isolation rates of the MCKT medium in culturing fecal specimens from healthy volunteers

From September 2019 to November 2019, 516 non-repeated fresh fecal specimens from healthy volunteers were collected and inoculated on an MCKT medium containing 4 μg/ml potassium tellurite, and a total of 86 colonies were isolated. All of these 86 cultured colonies were identified as KP strains by MALDL-TOF, the KP isolation rate was 16.7% (86/516).

#### The positivity rates of virulence gene of KP isolates in MCKT medium

Among the 86 strains of KP, the positive rate of *terW* gene was 100% (86/86), the positive rate of *rmpA* gene was 55.8% (48/86), and the positive rate of *iutA* gene was 52.3% (47/86). According to the definition of hvKP in this study, these strains can be classified into 55 strains of hvKP (55/86, 64.0%) and 31 strains of cKP (31/86, 36.0%).

#### The resistance characteristics of *terW*^+^ KP isolated by MCKT medium between healthy volunteers and patients

According to the 2019 CLSI antimicrobial susceptibility test standards (33), 86 strains of *terW*^+^ KP isolated from healthy people and 75 strains of *terW*^+^ KP isolated from clinical specimens were tested. There was no significance between the two groups in resistance rates of cefazolin, piperacillin/tazobactam, ceftriaxone, cefepime, imipenem, meropenem, ciprofloxacin and tobramycin (*p* > 0.05) (Table 5).

**Table 5:** The resistance rates of *terW*^+^ KP isolated by MCKT medium from healthy volunteers and inpatients.

**Antibiotic**	**Healthy volunteers** **(%, *n =* 86)**	**Inpatients** **(%, *n =* 75)**	***p*-Value**
Ampicillin	86 (100.0)	75 (100.0)	/^a^
Piperacillin/tazobactam	2 (2.3)	2 (2.7)	1.000
Cefazolin	8 (9.3)	11 (14.7)	0.293
Ceftriaxone	4 (4.7)	7 (9.3)	0.389
Cefepime	2 (2.3)	2 (2.7)	1.000
Imipenem	0 (0.0)	1 (1.3)	0.466
Meropenem	0 (0.0)	1 (1.3)	0.466
Ciprofloxacin	2 (2.3)	6 (8.0)	0.197
Tobramycin	3 (3.5)	4 (8.0)	0.369

a The p-value could not be calculated for both groups that were 100% resistant to ampicillin.

#### Distribution of major virulent capsular serotypes of *terW*^+^ KP isolated from feces of healthy volunteers by MCKT medium

Among the 86 *terW*^+^ KP strains, 27 strains were K1 (31.4%), 12 strains were K2 (14.0%), 2 strains were K5 (2.3%), 2 strain were K20 (2.3%), five strains were K54 (5.8%), and eight strains were K57 (9.3%). The total of K1, K2, K5, K20, K54, and K57 accounted for 65.1% (56/86).

**Figure 1:**
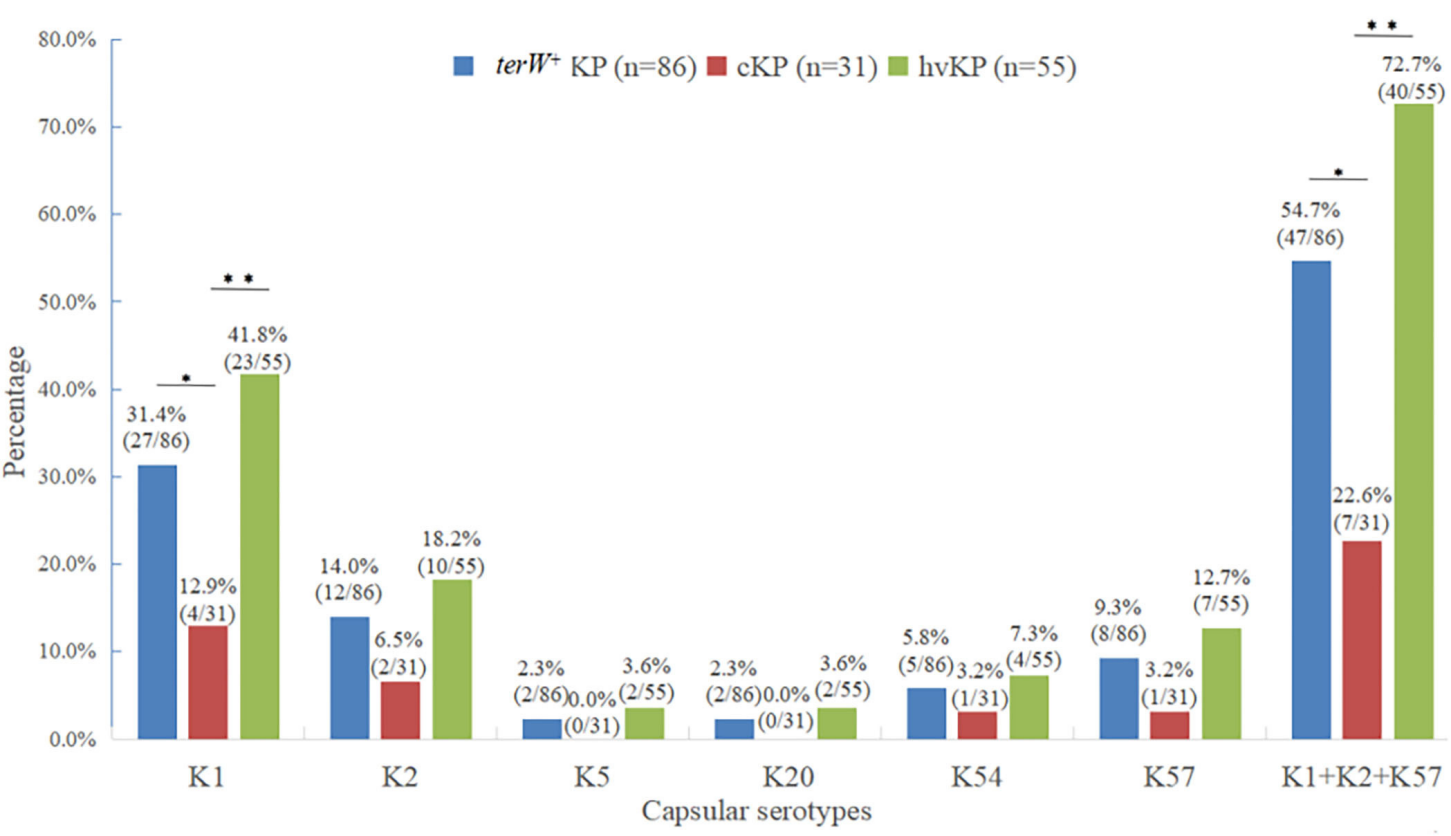
Distribution of major virulent capsular serotypes of *terW*^+^KP isolated from feces of healthy volunteers by MCKT medium (*P < 0.05, **P < 0.01, no asterisk: *P* > 0.05).

There was no significant difference between the capsular serotypes of *terW*^+^ KP and hvKP (*p* > 0.05). The total of K1, K2, and K57 in hvKP accounting for 72.7% (40/55) was significantly higher than that in cKP accounting for 22.6% (7/31) (*p* < 0.001). The positive rate of K1 in hvKP and *terW*^+^ KP was significantly higher than that in cKP (*p* < 0.05) (Figure 1).

## Discussion

Most hvKP infections are community-acquired infections, and it was generally believed that most hvKPs were sensitive to commonly used clinical antibiotics. In this study, most of the clinically isolated hvKPs were sensitive to commonly used clinical antibiotics, and the resistance rates were significantly lower than cKP, which was consistent with the previous studies (5, 37). But in the past years, hvKP has become a clinical challenge in China (4, 30), because the proportion of hvKP isolates increased from 25.5% to 54.5% within 2 years, and it resisted all testing resistant, except carbapenems and amikacin (32). Zhang et al. (38) first reported the detection of carbapenem-resistant hypervirulent KP (CR-hvKP). The CR-hvKP causes more severe disease and higher mortality than classical carbapenem-resistant Enterobacter strains (39). Due to a sharply rising proportion of hvKP isolates, and the high virulence coupled with high antimicrobial resistance of KP, an outbreak would be a disaster, so it is of great significance to monitor the antimicrobial susceptibility of hvKP.

The hvKP isolation rate was 42.9% in our study. In the previous data, the separation rates of hvKP ranged from 24.5% to 45.7% (3, 40), which was consistent with our data but different in sample size, region, and judgment criteria. Therefore, it is necessary to conduct a multi-center large-sample study to establish a clear hvKP identification marker to make the research data more accurate. In this study, the detection rates of *terW* gene of KP isolated from clinical specimens were 37.9%, and the detection rates of *terW* gene in hvKP were significantly higher than that in cKP, and it was speculated that the *terW* gene might be a potential virulence-related gene of hvKP.

The tellurite group (TeO${}_{3}^{-2}$) of soluble tellurite is highly toxic to most microorganisms and is considered to exert its toxicity as a strong oxidant by generating intracellular ROS (41). The presence of the *ter* operon increases the hydrogen peroxide-mediated oxidative stress response that increases tolerance to potassium tellurite by counteracting the effects of ROS, and the operon also prolongs the ability of specific strains to survive in macrophages to enhance the virulence of KP (17). The *ter* operon has also been found on other large conjugative plasmids like pTE53 plasmid from *E. coli* and pLVPK plasmid from KP CG43. All of the *E. coli* strains showing high tellurite resistance were founded to contain the *ter* operon. The genes composition of the *ter* operon in different E. coli strains were diverse, but all contained the *terW* gene. TerW protein is the first known functional *ter* gene product and binds specifically to the promoter region of the *ter* operon. TerW protein controls tellurite resistance levels by inducing overexpression of the *E. coli ter* gene (14). At present, there are many studies on the tellurite resistance of *E*. *coli*, but few studies on the tellurite resistance of KP. Previous studies have shown that the tellurite resistance of KP is related to the *ter* gene cluster (8, 16), but no one has reported the specific level of tellurite resistance of KP in China. In this study, the range of potassium tellurite resistance levels of KP was reported for the first time, and it was confirmed that the *terW* gene of KP was associated with high levels of potassium tellurite resistance, which was consistent with previous studies (8, 16).

Since *terW* is associated with the virulence of hvPK, in this study, a selective medium was manufactured by adding a certain concentration of potassium tellurite in the MacConkey medium. MacConkey medium is one of the most commonly used selection media in the clinical setting, which contains bile salts to inhibit gram-positive bacteria, lactose, and corresponding acid–base indicators to make lactose-fermenting bacteria appear pink colonies, and bacteria that do not ferment lactose appear colorless and transparent colonies. In this circumstance, KP can grow on MacConkey medium as large, moist, puffed pink colonies. In this study, 75 *terW*^+^ KP potassium tellurite resistance levels ranged from 8 to 128 μg/ml. The potassium tellurite resistance levels of 55 *terW*^−^ KP strains ranged from <1 to 8 μg/ml. Using MacConkey plates containing 4 μg/ml or 8 μg/ml potassium tellurite to screen *terW*^+^ KP, the sensitivity was 100 and 98%, respectively. As a screening test, a higher sensitivity is preferred. Therefore, the final concentration of potassium tellurite was set at 4 μg/ml. However, due to the heavy workload of MIC detection and statistical design, only 55 samples from 123 *terW*^−^ KP were randomly included. The accuracy of the MIC of potassium tellurite results of *terW*^−^ KP strains should be further improved. On MacConkey medium containing 4 μg/ml potassium tellurite, *terW*^+^ KP grows well in this medium. The colonies are large, moist, bulging, and pale pink, which are similar to that on ordinary MacConkey plates. Potassium tellurite is highly toxic to most microorganisms and can inhibit the growth of most bacteria, which can save a lot of labor work in the epidemiological study of fecal samples. Other pathogenic bacteria that are clinically resistant to tellurite, such as *Bacillus anthracis*, Yersinia, and enterohemorrhagic *E. coli* O157:H7, etc., (14, 16, 42, 43) were not identified in this study. These bacteria, as well as KP, can reduce the tellurite (TeO${}_{3}^{-2}$) of soluble tellurite to the less toxic Te element and deposit intracellularly to form black colonies (44). However, no black colonies were observed in KP grown on MacConkey plates containing 4 μg/ml potassium tellurite in this study, which may result from the relatively lower concentration of potassium tellurite and fewer black components produced by the fermentation of lactose.

MCKT medium culture method for screening *terW*^+^ KP has a good consistency and high sensitivity compared with PCR. The *terW*^+^ KP can grow on MacConkey plates containing 4μg/ml potassium tellurite. The lowest bacterial count was 10^1^–10^3^ CFU/ml. When inoculated with 10^1^ CFU/ml strains, 81.3% (61/75) of *terW*^+^ KP can grow on the surface of the medium, indicating that it has a good screening ability for low loads of bacteria, and it might cause little false-negative. Therefore, the MacConkey plate method containing 4 μg/ml potassium tellurite established in this study has the advantages of high sensitivity, strong specificity, rapidity, low cost, and simple operation, which is suitable for epidemiological investigation of large samples.

We applied this method to study fecal samples from healthy people in Fujian, China, in order to provide evidence for the prevention and control of *terW*^+^ KP infection. It is the first study to report the colonization rate of *terW*^+^ KP in the intestine of healthy people. The *terW*^+^ KP is closely related to the three clonal bacterial groups CG23, CG65, and CG86 that most commonly cause community-acquired purulent infections (16). The intestinal colonization of KP can cause transmission through household contact (45), suggesting that cross-infection also exists among healthy individuals. It shows that the *terW*^+^ KP carried by healthy people may be the source of infection of community-acquired purulent infection. Therefore, when *terW*^+^ KP is tested positive in a healthy individual, microbial flora transplantation can be used to resist colonization to achieve the purpose of prevention (26), and good hand hygiene can effectively prevent *terW*^+^ KP from spreading in households. The *terW-iutA-rmpA-silS* gene-derived locus is an important risk factor for abscess formation as well. It is a representative gene and derivative of the pLVPK plasmid and is considered to be a marker for detecting whether KP carries the pLVPK plasmid (7, 8, 24, 46). Therefore, the MCKT culturing method could also be used as the primary screening for the high-virulence plasmid pLVPK or a way to quickly obtain a large number of KP strains carrying pLVPK plasmids for scientific research.

The antimicrobial susceptibility testing results of 86 *terW*^+^ KP strains isolated from feces in 516 healthy people showed that the strains were sensitive to most antibiotics (resistance rates were less than 10%). There was no significant difference in the resistance rates of most antibiotics between 86 *terW*^+^ KP strains isolated from feces in healthy people and 75 *terW*^+^ KP strains isolated from 198 clinical specimens of inpatients. This result indicated that *terW*^+^ KPs screened by MCKT medium might be used as a clinical prediction of the drug susceptibility of hvKP, and provide guidance for the clinical medication of patients with a high risk of hvKP infection. However, our study needs more data on antibiotic resistance for further validation.

The capsular serotype is the most important protective antigen of KP. According to capsular serotype typing, KP can be divided into at least 79 capsular serotypes (K antigen) (47), among which K1, K2, K5, K20, K54, and K57 are considered to be the most common highly virulent capsular serotypes in clinical infections (48, 49). No studies have analyzed the capsular seroepidemiology of *terW*^+^ KP colonizing the gut. In this study, K1, K2, K5, K20, K54, and K57 were all detected, which also showed the diversity of intestinal microbiota, among which K1, K2, and K57 were the most common. In this study, 65.1% (56/86) of *terW*^+^ KPs belonged to the highly virulent capsular serotypes, which further proved that most of the *terW*^+^ KP was highly virulent. These results also confirmed from another aspect that the theory of using the tellurite resistance of *terW*^+^ KP to screen hvKP was reliable.

In conclusion, we prepared a medium culturing method to select the *terW*^+^ KP based on MacConkey supplied with potassium tellurite. It is a sensitive hvPK active screening method, which is economic, fast, and efficient, and can be used to study the colonization of *terW*^+^ KP strains in the intestine of a healthy population.

## Data availability statement

The original contributions presented in the study are included in the article/supplementary materials, further inquiries can be directed to the corresponding author/s.

## Ethics statement

The studies involving human participants were reviewed and approved by Branch from Research and Clinical Technology Application, Ethics Committee of the First Affiliated Hospital of Fujian Medical University (Approval No. MRCTA, ECFAH of FMU[2017]019). The patients/participants provided their written informed consent to participate in this study. Written informed consent was obtained from the individual(s) for the publication of any potentially identifiable images or data included in this article.

## Author contributions

XW, FZ, and BY designed the study. XW, FZ, JZ, SC, and BY had access to the raw data. XW and FZ contributed to the detection of the virulence genes, prepared MacConkey-potassium tellurite medium and the performance in detecting *terW*+ KP. XW, SC, and BY contributed to the detection of antimicrobial susceptibility. XW, FZ, JZ, and SC contributed to data analysis and interpretation. XW, FZ, and JZ contributed to the drafting of the article. BY attest that all listed authors meet authorship criteria and that no others meeting the criteria have been omitted. XW and BY are the guarantors. All authors provided final approval to publish.

## Funding

This work was supported by National Natural Science Foundation of China (No. 82172327), Fujian Province Health Technology Project (No. 2020CXA031), and Startup Fund for Scientific Research of Fujian Medical University (No. 2019QH1067).

## Conflict of interest

The authors declare that the research was conducted in the absence of any commercial or financial relationships that could be construed as a potential conflict of interest.

## Publisher's note

All claims expressed in this article are solely those of the authors and do not necessarily represent those of their affiliated organizations, or those of the publisher, the editors and the reviewers. Any product that may be evaluated in this article, or claim that may be made by its manufacturer, is not guaranteed or endorsed by the publisher.
